# Acute peripheral pulmonary embolism attributed to autoimmune haemolytic anaemia: a case report

**DOI:** 10.1186/s12872-020-01401-8

**Published:** 2020-03-04

**Authors:** Jing Xu, Liang Wang, Fadong Chen

**Affiliations:** Department of Cardiology, Shanghai East Hospital,Shanghai Tongji University School of Medicine, Shanghai, China

**Keywords:** Pulmonary embolism, Anemia, Hemolytic, Autoimmune

## Abstract

**Background:**

PE (pulmonary embolism) is a life-threatening complication rarely seen in the AIHA (autoimmune haemolytic anaemia) patients. Herein we reported a rare and serious AIHA-PE patient characterised by extensive peripheral pulmonary embolism on CTPA.

**Case presentation:**

A 59-year-old woman presented to our ED (emergency department) complaining of acute chest pain and dyspnea. During her presentation in ED she experienced a sudden syncope and soon developed CA (cardiac arrest). Laboratory studies showed a increase of CK-MB,troponin T,myoglobin and D-dimer. Computed tomography pulmonary angiography (CTPA) showed no large central or segment pulmonary emboli but increased RV (right ventricle)size,enlarged main pulmonary artery and invisible peripheral pulmonary artery. She was diagnosed with acute PE and alteplase was delivered intravenously. After thrombolytic therapy she remained hypotension and developed worsening anaemia. Detailed examination for anaemia revealed AIHA. She was discharged in a stable condition after 5 weeks with methylprednisolone and warfarin. Hb, D-dimer and transthoracic echocardiography showed complete recovery at 3-months follow up.

**Conclusion:**

PE attributed to AIHA is characterized by subsegment and distal pulmonary artery embolism which is easily neglected but always life-threatening. This case also highlights the PE as a secondary diagnosis should be evaluated comprehensively in order to identify the underlying pathogenesis.

## Background

AIHA is a rare autoimmune disease with an estimated annual incidence of 1 to 3 per 100,000 individuals [1]. VTE (Venous thromboembolism) such as PE is a life-threatening complication of AIHA which contributed to the death of 3–10% in these patients [2], thus how to timely identify and appropriately manage AIHA-PE patients remains a clinical challenge. Past studies had been confined to case reports or small patient series, while the clinical features of PE attributed to AIHA are undermined. In this report we present a rare case experiencing syncope and cardiogenic shock was finally diagnosed with AIHA-PE.

## Case presentation

**Fig. 1:**
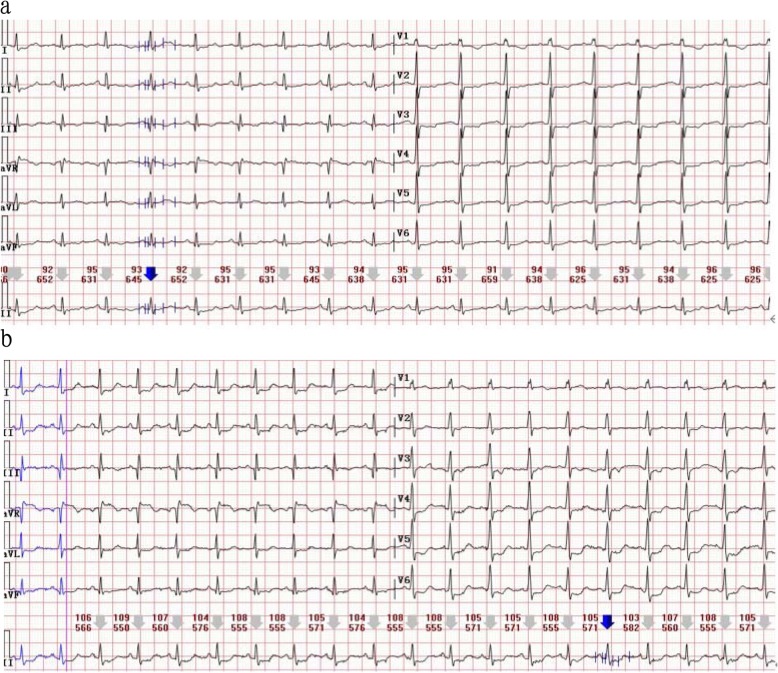
**a** Twelve-lead ECG at admission showing sinus rhythm (95 bpm) with ST depression in II,III,aVF,V4-V6. **b** Twelve-lead ECG at syncope showing ST-segment elevation in aVR and depression futher in II,III,aVF,V4-V6

A 59-year-old woman presented to our ED complaining of acute chest pain and dyspnea. She had vomited once,associated with fatigue and dizziness. She had no medical history. Vital signs at ED admission were stable: body temperature 37.3 °C; BP (blood pressure) 120/68 mmHg;heart rate 95 beats/min;respiratory rate 23 breaths/min;oxygen saturation 84.3% on room air. Cardiac examination showed high-pitched P2. Abdominal findings were unremarkable. There was no lower limbs edema. Initial blood analysis revealed Hb (haemoglobin) was 9.2 g/dl with white cell count 15.68× 10^9^/L,neutrophils 74.8% and platelet count 334× 10^9^/L; Cardiac enzymes test showed CK-MB was 1.96 ng/ml, troponin T was 0.046 ng/ml, myoglobin was 21.23 ng/ml and a high level of D-dimer (6.38 mg/l); A 12-lead ECG (Fig. 1a) confirmed a sinus rhythm with ST depression in II,III,aVF,V4-V6.

**Fig. 2:**
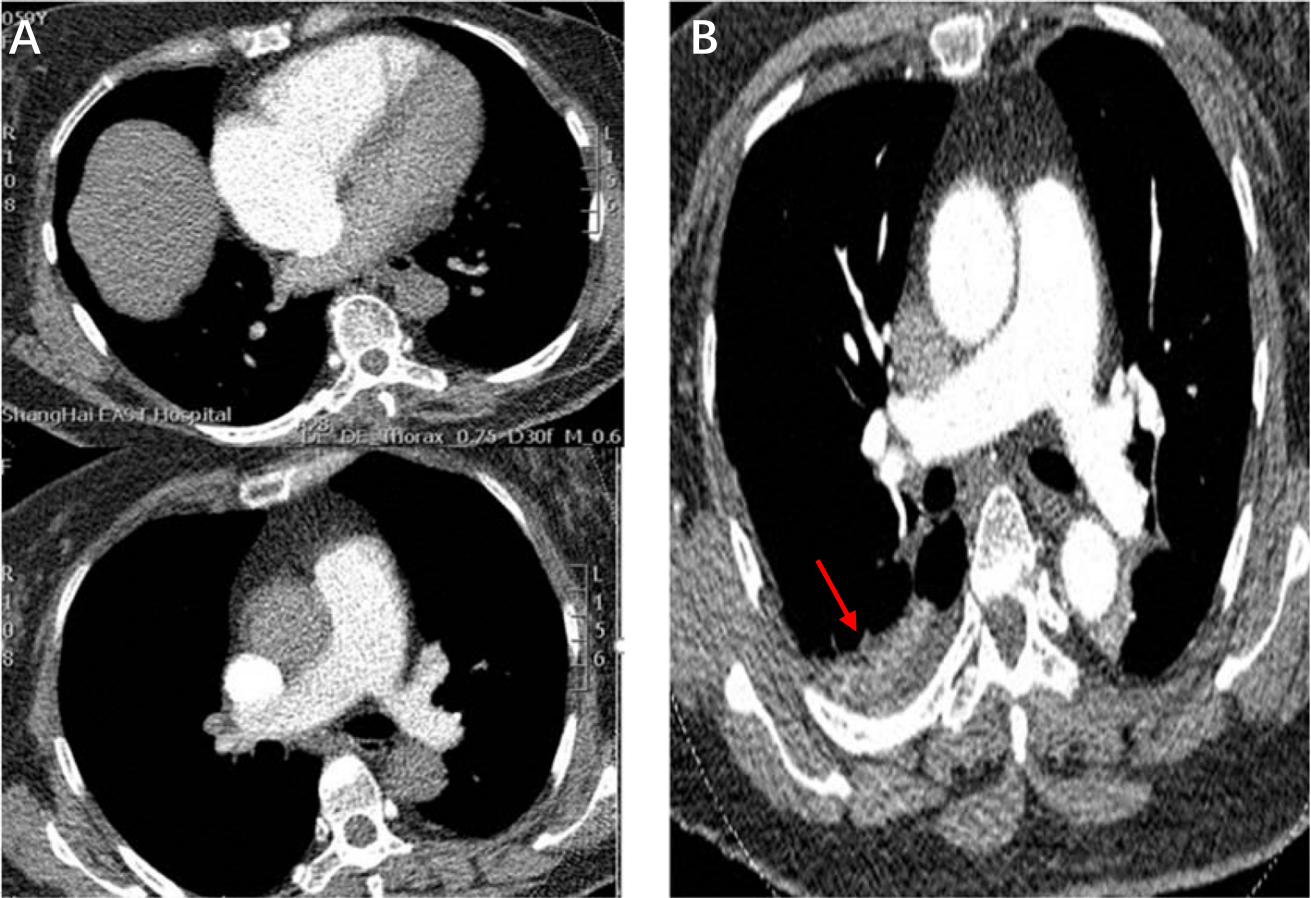
**a** CTPA at emergency department showing increased right ventricle size,flattened interventricular septum,enlarged main pulmonary artery and invisible peripheral pulmonary artery. No large central or segment pulmonary emboli was detected. **b** CTPA after thrombolytic therapy showing RV pressure overload ameliorated,peripheral pulmonary artery was visible and a little unilateral pleural effusion close to rib (highlighted by a red arrow)

**Fig. 3:**
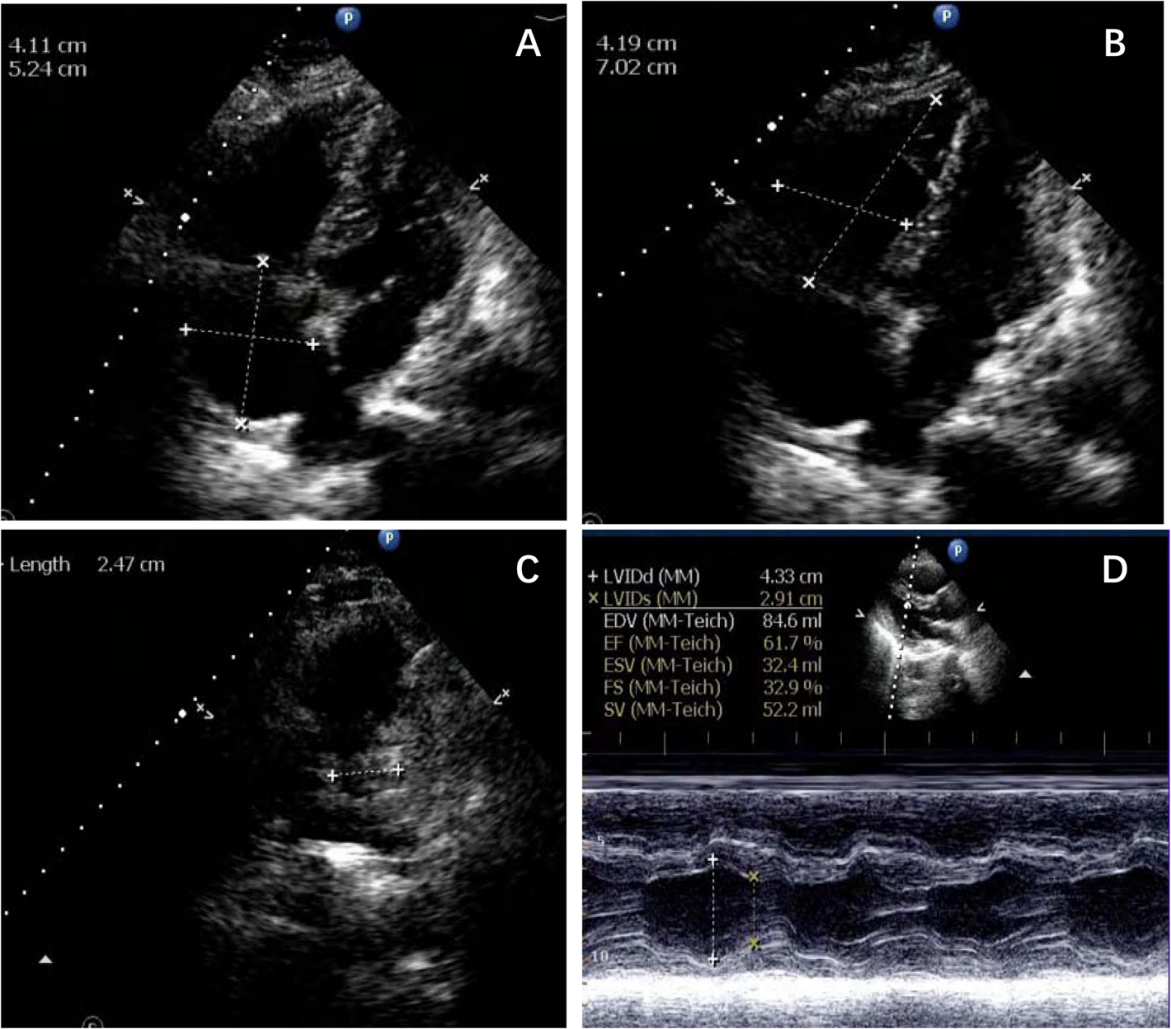
TTE at bedside after thrombolytic therapy. **a** Dilated right atrium (41mm×52mm). **b** Dilated right ventricle (42mm×70mm). **c** Pulmonary artery diameter (25mm) was measured by TTE. **d** Left ventricular size and ejection fraction were measured by TTE

About 6 h after admission,she suddenly developed syncope on the way to the toilet. Her heart rate increased to 100-130beats/min with hypotension (BP 80–90/55-60 mmHg) and low oxygen saturation (85–90% on nasal oxygen inhalation). Reexamined 12-lead ECG (Fig. 1b) exhibited ST-segment elevation in aVR and depression futher in II,III,aVF,V4-V6. Laboratory studies showed a further increase of CK-MB (3.76 ng/ml),troponin T (1.75 ng/ml),myoglobin (47.28 ng/ml) and D-dimer (7.36 mg/l). To exclude an acute PE, emergent CTPA (Fig. 2a) was performed showing increased RV size,flattened interventricular septum,enlarged main pulmonary artery and invisible peripheral pulmonary artery. No large central or segment pulmonary emboli was detected. She suddenly developed bradycardia and following CA on the way to the cardiac care unit for further treatment. Given the patient’s previous findings (ECG,CTPA,laboratory studies) and clinical status, the diagnosis of acute PE (high risk) was established, 50 mg alteplase was delivered intravenously within 1 h and cardiopulmonary resuscitation was promptly initiated. Meanwhile,she was intubated receiving mechanical ventilation. She recovered consciousness 2 h after uninterrupted CPR. At the second day,her clinical symptoms markedly ameliorated, heart rate and oxygen saturation recovered to 70-80 bpm and 95% without mechanical ventilation,respectively. The repeat CTPA (Fig. 2b) revealed RV size diminished and peripheral pulmonary artery was visible. TTE (transthoracic echocardiogram) (Fig. 3) revealed dilated right heart with increased pulmonary arterial systolic pressure (PASP 40 mmHg). She remained hypotension and developed worsening anaemia of Hb 57 g/l without any bleeding. Further laboratory examination aiming at anaemia revealed the percentage of reticulocyte was 9.7%, absolute reticulocyte count was 0.186 × 10^12^/L, total bilirubin was 40.9umol/L with unconjugated bilirubin 28.7 umol/l,lactate dehydrogenase was 6669.70 U/L. In addition, the patient’s direct antiglobulin test was reactive for IgG and was negative for complement. Bone marrow examination, a search for lymphoproliferative disorders and immune diseases were all negative.

**Fig. 4:**
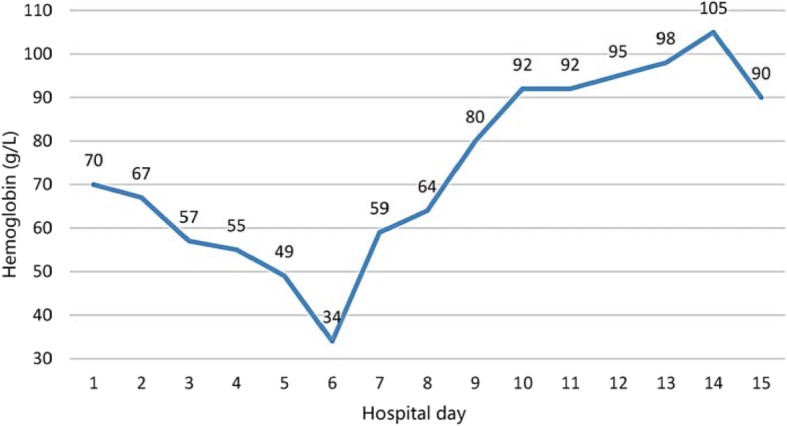
The Change of hemoglobin throughout the course of hospitalization. At HD5, the patient was transfused with 2 units of Washed Red Blood Cells. Methylprednisolone and immunoglobulin were initiated on HD7

On the fifth day of hospitalization,she was transfused with 2 units of Washed Red Blood Cells due to decreased Hb of 49 g/l,which fell further to 34 g/l after transfusion therapy. Intravenous corticosteroids (methylprednisolone 60 mg bid) plus immunoglobulin (IVIg 10 g qd) were added to her regimen. The anaemia improved progressively during the following days (Fig. 4).

She was discharged in a stable condition after 5 weeks with oral methylprednisolone (40 mg qd) and warfarin (2.5 mg qd). At 3-months follow up,the patient was asymptomatic and presented a complete recovery with Hb 141 g/l and normal D-dimer. TTE showed a complete disappearance of initial abnormalities. The patient’ current medication including methylprednisolone (4 mg qd) and warfarin (2.5 mg qd).

## Discussion and conclusion

AIHA is an acquired haemolysis caused by the host’s immune system acting against its own red cell antigens. It can occur at any age but incidence rises with increasing age. AIHA is divided into warm antibody (wAIHA), cold antibody-mediated (cAIHA) or mixed AIHA. Approximately half are primary (idiopathic) AIHA and half are secondary to associated disorders,including neoplasia, infection,immune dysregulation or drug induced [3].

The increased risk of PE associated with AIHA is an increasing matter of concern due to significant morbidity and mortality. As early as the 1960s, a review of 47 patients found the most common cause of death was PE [4]. However, the clinical features of PE attributed to AIHA are undermined. In 2015,Andrew Woodson [5] reported a 52-year-old man presenting as progressive fatigue, weakness and palpitations was diagnosed with AIHA-PE.He experienced profound haemodynamic compromise while no large central emboli,only small pulmonary emboli in bilateral subsegmental arteries was observed on CTPA. Our case initially presenting with chest pain and syncope was considered as PE due to increased RV loading, broadened main pulmonary artery and invisible peripheral pulmonary vessels on CTPA. In addition, the diagnosis of PE (high risk) could be met in this patient for typical clinical presentations (chest pain,syncope and CA),the laboratory tests(D-dimer, oxygen saturation, troponin T) and echocardiogram (dilated right heart and increased PASP). Our case indicated that PE attributed to AIHA is characterized by subsegment and distal pulmonary artery embolism, which is easily neglected but always life-threatening,thus needing us to detect and manage early to improve clinical outcomes.

This case report highly suggested comprehensive evaluation for the cause of PE in patients with first occurrence. Major risk factors for PE include surgery,active cancer, immobility, trauma or fracture, pregnancy, and estrogen therapy. In our case,it is paradoxical that after thrombolytic therapy, the patient remained hypotension while other evidence confirmed thrombolytic is effective as the clinical symptoms markedly improved, oxygen saturations and heart rate were normalized, D-dimer rised rapidly and following fell gradually, RV pressure overload ameliorated on CTPA. Besides, the patient admitted in ED with low level of hemoglobin (92 g/l) and decreased futher (57 g/l) without any hemorrhage after thrombolytic therapy,which intrigue us to investigate the real etiologe of PE. Primary wAIHA is finally diagnosed based on further laboratory examination. This case highly suggested the patients presenting with PE should be systematically investigated with scrutiny in order to explore the potential diseases.

The AIHA patients complicated by PE should be treated aggressively as early as possible. When suffering from haemodynamic compromise, the patients should be administered with thrombolytics immediately [6]. First line treatment for primary wAIHA is oral prednisolone. Intravenous methylprednisolone and immunoglobulin may be considered in severe, life-threatening cases [7]. In our case,intravenous methylprednisolone and IVIg were added to her regimen when Hb fell further after transfusion. Transfusion should be treated with caution due to the risk of hemolytic transfusion reactions caused by alloantibodies of AIHA. Given the higher risk of thrombosis, long-term anticoagulation should be advised [8]. Despite the improved safety of Non-vitamin K antagonist oral anticoagulants (NOACs) compared with Vitamin K antagonists (VKAs) for extended anticoagulation of acute PE, treatment with NOACs is not without risk [9]. This patient was administered with warfarin since VKAs have been the gold standard in oral anticoagulation. NOACs may be an alternative to VKAs in this setting but there is no evidence-based practice [7, 9].

In conclusion, We present a rare case of previously healthy female patient experiencing a sudden syncope and following cardiac arrest for PE attributed to AIHA. Upon encountering suspected high-risk PE based on clinical probability,which is characterized by peripheral pulmonary emboli and severe anemia, AIHA should be considered. Close attention as well as timely treatment play a crucial role.

## Data Availability

The datasets used in the case are available from the corresponding author upon reasonable request.

## Abbreviations

AIHA: Autoimmune haemolytic anaemia
BP: Blood pressure
CA: Cardiac arrest
CTPA: Computed tomography pulmonary angiography
ED: Emergency department
Hb: Haemoglobin
NOACs: Antagonist oral anticoagulants
PE: Pulmonary embolism
RV: Right ventricle
TTE: Transthoracic echocardiogram
VKAs: Vitamin K antagonists
VTE: Venous thromboembolism
